# Prostatic artery embolization: Progress and prospect

**DOI:** 10.1016/j.jimed.2020.03.003

**Published:** 2020-03-28

**Authors:** Li Cui, Yanhua Bai, Jinlong Zhang, Bing Yuan, Xiuqi Wang, Yan Wang, Feng Duan, Maoqiang Wang

**Affiliations:** Department of Interventional Radiology, The General Hospital of Chinese People’s Liberation Army, Beijing, 100853, China

**Keywords:** Benign prostatic hyperplasia, Prostatic artery embolization, BPH, PAE

## Abstract

Prostate artery embolization is a well-known and promising treatment for benign prostatic hyperplasia, with the quantum leaps of research in medicine. We aim to provide an up-to-date review of the novel technique, including large retrospective studies and randomized control trials, ends with discussions of advantages and disadvantages of this minimally invasive technique.

Benign prostatic hyperplasia (BPH) is a common disease in middle-aged and elderly men, with an incidence of >50% in those aged over 50 years and >90% in those aged over 80 years.^1^ BPH often induces lower urinary tract symptoms (LUTS) such as bladder outlet obstruction and dysuria, which further damage the bladder and kidneys. Symptomatic BPH can seriously affect patient health and quality of life.^2^ Current common treatments for BPH include drug therapy, open prostatectomy, and transurethral resection of the prostate (TURP), but each has certain limitations. Prostatic artery embolization (PAE) is a novel minimally invasive treatment for BPH with the advantages of high safety, minor trauma, short hospital stay, and repeatability and has become a hot research topic in recent years.^2^

## 1 Evolution of PAE

### 1.1 Research advances in China

In 2002, Hao et al described 15 cases of BPH successfully treated with PAE,^3^ the first report on PAE worldwide. In 2005, Cheng et al^4^ first reported that PAE using particles of 150–250 ​μm could decrease prostate volume and eliminate compression on the urethra; thus, PAE has become a promising non-surgical treatment strategy for BPH. However, the authors failed to compare the efficacies of embolic agents of different particle sizes.

In 2006, Gao et al^5^ reported 12 cases of BPH treated with PAE, in which the prostate volume was reduced by a mean 51% and no serious complications were noted, suggesting that PAE is a treatment with minor trauma, good efficacy, high safety, and fewer complications. However, as only 12 cases were presented, their conclusions must be further verified in studies with larger sample sizes.

In 2008, Gao et al^6^ reinvestigated the origin of prostate blood-supplying arteries and identified the predominant arteries using digital subtraction angiography (DSA). During PAE in 72 cases (237 arteries), the inferior vesicle artery (IVA; n ​= ​69), internal iliac artery (IIA; n ​= ​63), internal pudendal artery (IPA; n ​= ​52), inferior rectal artery (n ​= ​29), and superior vesicle artery (n ​= ​14) were observed. There were 63 predominant arteries, mainly those originating from the IIA (n ​= ​37), IVA (n ​= ​20), IPA (n ​= ​6), and inferior rectal artery (n ​= ​2). These findings demonstrated that arterial angiography has important clinical value for guiding PAE. This was one of the earliest studies to describe the anatomical origins of the arteries within the prostate and provide directions for subsequent studies.

In 2010, Gao et al^7^ assessed the clinical efficacy of PAE for treating BPH in 47 cases and reported that International Prostate Symptom Score (IPSS), quality of life (QoL), Qmax, and residual urine were markedly improved after the procedure, while prostate volume was significantly reduced by a mean 41.8% and the efficacy rate of PAE reached 89%. These results reconfirmed that PAE could be a novel minimally invasive treatment for BPH. However, these articles were published in Chinese-language journals and the procedure itself had some technical problems, which restrict its global applications.

In 2013, Deng et al^8^ reported 16 BPH cases treated with PAE (PAE group) compared with 35 cases treated with TURP (control group). The PAE group was superior to the control group in terms of the improvement of various indicators and showed no major complications. This was the first study to compare PAE with traditional surgical techniques, and this non-inferiority-controlled study highlighted the clinical value of PAE.

In 2014, Gao et al^23^ conducted a prospective randomized and controlled clinical trial (RCT) that compared PAE with TURP in the treatment of patients with BPH. A total of 114 patients were randomly assigned to undergo PAE (n ​= ​57) or TURP (n ​= ​57). The TURP group had better clinical efficacy, while the PAE group had higher rates of adverse events and complications (e.g., postembolization syndrome). The low efficacy and high incidence of complications in the PAE group might be attributed to the suboptimal operations by interventional radiologists, large particle sizes of the embolic materials, and randomization of unilateral or bilateral PAE.

In 2016, Wang et al^16^ reported a single-center study on differentiating the prostatic arteries (PAs) using DSA and cone-beam computed tomography (CT). A total of 148 patients were enrolled and underwent DSA and cone-beam CT before the embolization. The PAs most frequently originated from the common gluteal-pudendal trunk and the superior vesicular artery, followed by the anterior division of the IIA and the IPA. Anastomoses to adjacent arteries were detected in 67 (22.6%) of 294 pelvic sides. The authors concluded that DSA combined with cone-beam CT can accurately determine the anastomosis of PAs and avoid the complications of misembolization and ectopic embolization and can be used in the planning of preoperative treatment.

In 2018, Wang et al^9^ investigated the impact of different polyvinyl alcohol (PVA) particle sizes on the therapeutic effect of PAE in a prospective RCT. A total of 120 BPH patients were enrolled. Group A underwent embolization with 50 μm and 100 μm PVA particles in the distal and proximal PAs, respectively. Group B used 100 μm PVA particles alone. The therapeutic effect was reportedly better in group A than in group B, and no severe complications occurred in either group. This study provided a novel embolization technique and shed new light on future research on PAE.

In 2019, Yuan et al^10^ performed a retrospective study of 8 BPH patients accompanied by bladder fistula treated with PAE. Intraoperatively, two embolic agents (50 and 100 μm particles) were used with a success rate of 100%. Six patients were followed up for 12 months, and IPSS, QoL, and prostate volume were significantly improved after the procedure (*P* ​< ​0.05); PSA increased 24 ​h after the embolization and decreased to the baseline level after 1 month (*P* ​> ​0.05). Standardized Infection Ratio (SIR) grade A complications were found in three patients, but no severe complications were noted. PAE was a safe and effective treatment for BPH accompanied by bladder fistula. This study also demonstrated that PAE could be applied for patients with BPH as well as those with bladder fistula.

In 2019, Zhang et al^24^ evaluated the role of contrast-enhanced magnetic resonance (MR) angiography in PAE for BPH patients. A total of 100 BPH patients undergoing PAE were included and randomly assigned to two groups. Group A (n ​= ​50) underwent PAE directly, and group B (n ​= ​50) underwent MR angiography prior to PAE. MR angiography identified PAs with a sensitivity of 91.5% and a positive predictive value of 100%. Operating and fluoroscopy times were significantly shorter in group B than in group A. Additionally, the radiation dose was markedly lower in group B than in group A. This study indicated that contrast-enhanced MR angiography could accurately visualize PA anatomy, thus shortening the PA embolization time and decreasing the radiation exposure.

#### Research advances in foreign countries

At the end of the 20th century, there were individual reports on the treatment of BPH patients accompanied by hematuria with PAE.^11^ However, these case reports did not attract much attention from our international peers.

In 2010, Carnevale et al^12^ reported two cases of BPH accompanied by acute urinary retention treated with PAE. The prostate volume was markedly reduced over 6 months of follow-up, which confirmed the efficacy of PAE for the first time worldwide.

In 2011, Sun et al^13^ reported a prospective animal experimental study. After the animal models of prostatic hyperplasia were established, the animals were divided into PAE and untreated groups. The prostate volume was significantly smaller in the PAE group than in the untreated group, suggesting that PAE was a safe and effective treatment that could be applied in the clinical setting. This was the first report on the efficacy of PAE in animal models.

In 2013, Pisco et al^14^ reported a prospective study of PAE treatment in 89 patients with BPH. During the 6- and 12-month follow-up periods, the symptom improvement rates were 78% and 76%, respectively, indicating that PAE could significantly reduce prostate volume and improve the symptoms associated with lower urinary tract obstruction.

In 2016, Pisco et al^15^ reported 630 cases of moderate to severe symptomatic BPH treated with PAE and showed that the medium- (1–3 years) and long-term (3–6.5 years) clinical effectiveness rates were 81.9% and 76.3%, respectively. No complications such as urinary incontinence or sexual dysfunction were found. This study had the largest sample size and longest follow-up duration.

In 2018, Ray AF et al^16^ compared PAE with TURP in a retrospective multicenter study in the United Kingdom and found that PAE was highly effective at alleviating symptoms and improving quality of life. Compared with TURP, PAE had the advantages of being performed in outpatient clinics with a significantly shortened length of hospitalization and recovery time. In contrast, PAE was more dependent on radiologist experience and required the use of imaging equipment. In Western countries including the United Kingdom, however, TURP remains the recommended treatment for BPH and the clinical value of PAE must be further recognized, which may be because most of the studies were initiated by urologists and focused on the superiority of surgical treatment options.

## 2 Current problems with PAE

### 2.1 PA anatomy

A key step of PAE is to identify PAs. According to classical anatomy, humans have no independent PAs; most originate from the inferior bladder artery.^2^ With advancements in imaging technology, *in vivo* anatomical studies on PAs demonstrated obvious advantages over autopsy specimens.^17^ Based on imaging techniques, Carnevale et al^18^ categorized PAs into five types: type I, IVA originating from anterior division of the IIA in a common trunk with SVA; type II, IVA originating from the anterior division of the IIA inferior to the SVA; type III, IVA originating from the obturator artery; type IV, IVA originating from the IPA; and type V (others), less common origins including the internal paragenital arteries, posterior trunk of the IIA, and inferior abdominal wall arteries.

Wang et al^17^ evaluated PA anatomy by cone-beam CT in conjunction with DSA in 148 Chinese BPH patients. There were a total of 318 ​PAs. One PA was identified in 274 versus two PAs in 22 pelvic sides. PA origins included the superior vesicular artery (37.1%), anterior division of the IIA (31.1%), IPA (24.2%), obturator artery (4.7%), and middle rectal artery (2.8%). Bilateral symmetry of the PA origin was present in 18 (12.2%) patients. In 67 (22.6%) pelvic sides, anastomoses to adjacent arteries were observed.

### 2.2 Selection of embolic materials

The proper selection of embolic materials and particle sizes is a key factor affecting PAE efficacy. Commonly used embolic materials currently include a) non-spherical embolic particles such as PVA particles and gelatin sponge and b) spherical microspheres such as PVA microspheres.

Bilhim et al^19^ compared the values of 100–300 ​μm non-spherical PVA particles versus 300–500-μm PVA microspheres in PAE and found no statistically significant difference in clinical efficacy, which might be due to the use of different particle sizes.

In another study by Hwang et al.*,*^20^ embolization was performed using non-spherical PVA particles (250–355 ​μm) in four BPH patients versus spherical microspheres (300–500 ​μm) in four BPH-induced LUTS patients. The microspheres showed greater prostatic volume reduction than non-spherical PVA particles. However, further investigations are needed to verify these findings due to the small sample size of this study, and no consensus has been reached regarding the preferred sizes of microsphere particles.

### 2.3 Prevention and management of complications after PAE

The incidence of PAE-related complications is low. Most complications are self-limiting and require no special treatment. Most of the complications are caused by the incidental entry of embolic materials into the blood vessels of adjacent organs, leading to ischemia of the penis, bladder wall, and rectum^14^^,^^21^ that can be spontaneously resolved after timely and effective management.

## 3 Challenges and prospects

In recent years, urologists in China and abroad have actively compared PAE with classic surgical treatments. In the UK-ROPE study, Ray et al^15^ enrolled 305 patients (PAE for 216, TURP for 89), and PAE was significantly more effective than TURP at alleviating symptoms, improving QoL, and shortening hospital stay. However, PAE requires high levels of technical expertise and rich clinical experience. Thus, PAE can replace classic surgical treatments only in selected cases. A meta-analysis performed by Jiang et al^22^ included 506 patients from four studies performed between May 1998 and May 2018, and the TURP group had significantly higher clinical effectiveness. Therefore, the authors concluded that TURP was superior to PAE.

Despite some controversy, the safety and near- and medium-term efficacies of PAE as a novel minimally invasive procedure for BPH have been well demonstrated; in particular, for elderly patients who refuse or cannot tolerate surgery, PAE has unique advantages. Notably, the well-known and influential studies mentioned above were performed by urologists. Thus, more prospective multicenter RCTs are required to further investigate the clinical value of PAE. It has been well accepted that PAE is a unique treatment for BPH or prostate tumors accompanied by refractory bleeding that deserves more widespread promotion in clinical practice.

## Declaration of interests

The author declare that they have no known financial interests or personal relationships that could have appeared to influence the work reported in this paper.
